# DPP-4 Inhibitor Reduces Central Blood Pressure in a Diabetic and Hypertensive Patient: A Case Report

**DOI:** 10.1097/MD.0000000000001068

**Published:** 2015-07-13

**Authors:** Luciana Neves Cosenso-Martin, Luiz Tadeu Giollo-Junior, José Fernando Vilela-Martin

**Affiliations:** From the Department of Internal Medicine, Medical School of São José do Rio Preto (FAMERP), Hypertension Clinic of FAMERP and Hospital de Base, Ave Brig Faria Lima 5416, São José do Rio Preto, SP, Brazil.

## Abstract

Hypertension and type 2 diabetes mellitus (DM) are among the main risk factors for the development of cardiovascular disease. Pharmacotherapy for DM should not only improve blood glucose control, but also provide beneficial glucose-independent cardiovascular effects. The central systolic blood pressure (SBP) has become more important than the brachial SBP in the assessment of cardiovascular risk.

This case report describes the effect of vildagliptin, a dipeptidyl peptidase-4 (DPP-4) inhibitor, on the central SBP in a 54-year-old woman with hypertension and DM. She was submitted to applanation tonometry (AT) before and after vildagliptin association. AT of the radial artery is a non-invasive method that indirectly assesses arterial stiffness by calculating the central SBP and the augmentation index (AIx).

After 3 months of follow-up using vildagliptin, central SBP and AIx were improved. Moreover, she presented better glycemic control.

This case suggests an effect of DPP-4 inhibitor on arterial stiffness parameter (central SBP) in a hypertensive and diabetic patient, which shows a glucose-independent beneficial cardiovascular effect of this group of drugs.

## INTRODUCTION

Hypertension and type 2 diabetes mellitus (DM) are among the main risk factors for the development of cardiovascular disease (CVD). In fact, DM is associated with a 2 fold higher risk for CVD.^1^ Endothelial dysfunction, associated with DM and hypertension, is considered an early marker of vascular complications and a pathophysiological determinant of atherogenic processes.^2^ Pharmacotherapy for DM should not only improve blood glucose control, but also provide beneficial glucose-independent cardiovascular effects. Dipeptidyl peptidase-4 (DPP-4) inhibitor is an incretin-based drug approved for the treatment of DM.^3^ This medication reduces the breakdown of glucagon-like peptide 1 (GLP-1), thereby increasing circulating GLP-1 levels, improving metabolic control by increases in insulin secretion, followed by decreases in glucagon secretion.^3^ Pharmacotherapy based on the GLP-1 system provides beneficial effects on the endothelium.^4–6^ Recently, several methods have been developed to assess endothelial function, and predict the presence or absence of coronary heart disease (CHD).^7^ Applanation tonometry (AT) of the radial artery is a noninvasive method that indirectly assesses arterial stiffness by calculating the central blood pressure (BP) and the augmentation index (AIx).^8,9^ The AIx is associated with cardiovascular risk, and is a predictor of CHD development.^9^ More recently, the central systolic blood pressure (SBP) of the aortic or carotid arteries has become more important than the brachial SBP in the assessment of cardiovascular risk.^10^ This case report describes a possible pleiotropic action of a DPP-4 inhibitor (vildagliptin) on the central SBP assessed by AT in a hypertensive diabetic woman. This pharmacological class seems to have action in reduction of central BP and arterial stiffness. Thus, we justify the possible pathophysiological mechanisms involved in the association between hyperglycemia, endothelial dysfunction, and vascular stiffness, besides how the GLP-1 system provides beneficial effect on the endothelium.

## CASE REPORT

The patient was a 54-year-old white woman with a 4-year history of hypertension and DM. She was taking metformin (850 mg/d), pioglitazone (30 mg/d), simvastatin (10 mg/d), amlodipin (5 mg/d), and enalapril (10 mg/d); however, she did not adhere to a diet to control the diabetes. Her physical examination revealed BP: 123/85 mm Hg, heart rate: 78 bpm, body mass index (BMI): 29.1 kg/m^2^, and waist circumference: 91 cm. She had no abnormalities of the heart, lungs, or abdomen. According to complementary exams, the patient had poor diabetic control with glycosylated hemoglobin (HbA1c): 11.2%; however, microalbuminuria and other biochemical parameters were normal.

The patient received guidance to modify her lifestyle including diet and exercise, and vildagliptin (100 mg/d) was added to her drug regimen. The patient was submitted to examinations of the radial artery using a commercially available automated AT system (HEM-9000AI; Omron Healthcare Co., Ltd, Kyoto, Japan) before receiving vildagliptin and 3 months after to evaluate the drug's effect on the central SBP and AIx. This examination was performed in a quiet controlled environment (temperature between 21°C to 24°C), between 8 am and 10 am, after the patient was rested for at least 10 minutes sitting with the legs uncrossed, the bladder empty, and away from acute stressors.^9^ All measurements were performed after at least 8-hour fasting. The patient was instructed to fast starting the night before testing and to refrain from ingesting alcohol or caffeine. The AT equipment uses a radial ultrasonic transducer and cuffs with the correct size for the arm circumference as recommended by the guidelines to evaluate BP. Pulse wave analyses were performed at least 3 times and the mean of measurements was calculated. Variations of BP between the measurements should not be >5%.^9^

**FIGURE 1:**
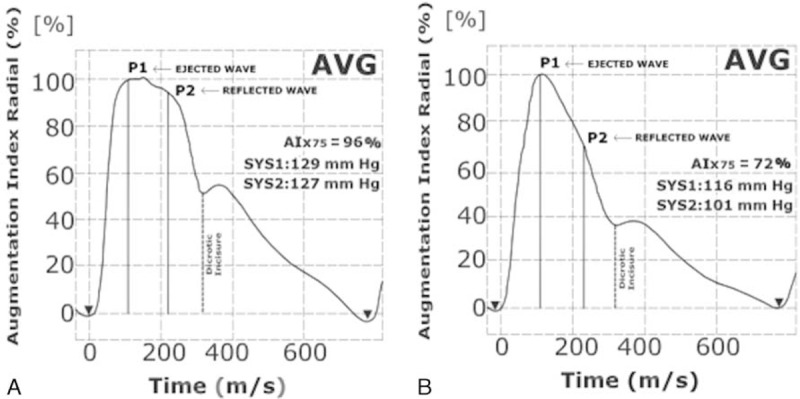
Waves and values of blood pressure measured with the applanation tonometry. (A) Before association of vildagliptin and metformin, Sys1 was 129 mm Hg. Sys2 and adjusted AIx for a heart rate of 75 bpm (AIx75) were 127 mm Hg e 96%, respectively. AIx75 under 100% is considered normal. Sys2 (central) was only 2 mm Hg lower than the peripheral systolic blood pressure and this difference should be about 15 to 20 mm Hg or 15% lower than Sys1. (B) After 3 months of vildagliptin and metformin association, Sys1 was 116 mm Hg, Sys2 was 101 mm Hg, and AIx75 was 72%. Both Sys2 and AIx presented reduction and Sys2 had become 15 mm Hg lower than the Sys1. AIx = augmentation index, AIx75 = adjusted augmentation index for a heart rate of 75 bpm, Sys1 = peripheral systolic blood pressure, Sys2 = central systolic blood pressure.

At the end of the 3-month follow-up period, the patient had good adherence to diet and exercise and had lost 3 kg, presenting with a BMI of 27.5 kg/m^2^, and an office BP of 100/70 mm Hg. The control of the diabetes was better with a level of HbA1c of 7.2%. Central SBP and AIx were lower than in the baseline results. Before the association of vildagliptin and metformin, the peripheral SBP was 129 mm Hg during the AT. The central SBP (aortic) and adjusted AIx for a heart rate of 75 bpm (AIx75) were 127 mm Hg and 96%, respectively (Figure 1A). After receiving vildagliptin for 3 months, the peripheral SBP was 116 mm Hg, central SBP was 101 mm Hg, and AIx75 was 72% (Figure 1B) during the AT. So, there were reductions in both the central SBP and AIx with central SBP becoming 15 mm Hg lower than the peripheral SBP. I affirm that the patient has given the informed consent for publication of the case.

## DISCUSSION

DM is associated with a 2 fold higher risk for CVD.^1^ Preventive strategies are important to reduce cardiovascular risk. An assessment and stratification of cardiovascular risk should be considered in order to identify individuals at higher risk of developing cardiovascular events. Thus, the adoption of preventive and pharmacological strategies is important to delay possible cardiovascular complications. In recent years, several methods have been developed to predict cardiovascular risk, including the use of ultrasound to measure the carotid intima-media thickness (cIMT) and the aortic pulse wave velocity, and computed tomography to identify and quantify calcification of coronary arteries, among others.^7^

In this case, the AT of the radial artery was used to assess vascular disease. In young healthy individuals, the central SBP (aortic) should be between 15 and 20 mm Hg lower than the peripheral SBP (brachial). Before DPP-4 inhibitor use in this patient, the central SBP was only 2 mm Hg lower than the peripheral SBP. After 3 months in use of vildagliptin, the difference between the central SBP and peripheral SBP increased to 15 mm Hg. This finding might be explained by the effect of the drug on the arterial tree, that is, it may represent a reduction in arterial stiffness. The central SBP and AIx are strongly correlated. In this case, AIx also improved after DPP-4 inhibitor use, even though both were normal at baseline. AIx is associated with cardiovascular risk as it identifies the presence or absence of CHD, and thus it is also considered a marker of vascular stiffness.^9^

According to European Society of Hypertension Guidelines, the evaluation of asymptomatic target organ damage (TOD) is crucial in determining the cardiovascular risk in hypertensive individuals. Among the TOD to be assessed is vascular stiffness, which can predict cardiovascular mortality independently of the stratification scores of cardiovascular risk.^11^ The evaluation of central hemodynamic, including carotid-femoral pulse wave velocity, central BP, and AIx, is important to determine the true cardiovascular risk. The Guidelines state that the measurement of central hemodynamic parameters is of great interest to mechanistic analyses in pathophysiology, pharmacology, and therapeutics, and that the central BP represents the true load imposed on heart, brain, kidney, and large arteries instead of the brachial BP.^11^ Thus, both central BP and AIx present better predictive value for cardiovascular events and for the differential effect of antihypertensive drugs compared to the brachial BP.^11,12^

Endothelial dysfunction is considered an early marker of vascular complications and a pathophysiological determinant of the atherogenesis that occurs in the early stages of CHD.^2^ Exposure to cardiovascular risk factors may trigger the atherosclerotic process that evolves with oxidative stress and nitric oxide (NO) inactivation.^13^ Endothelial dysfunction comprises a number of functional alterations such as impaired endothelium-dependent vasodilatation, impaired barrier function, and higher inflammatory and pro-coagulant activity.^13^

**FIGURE 2:**
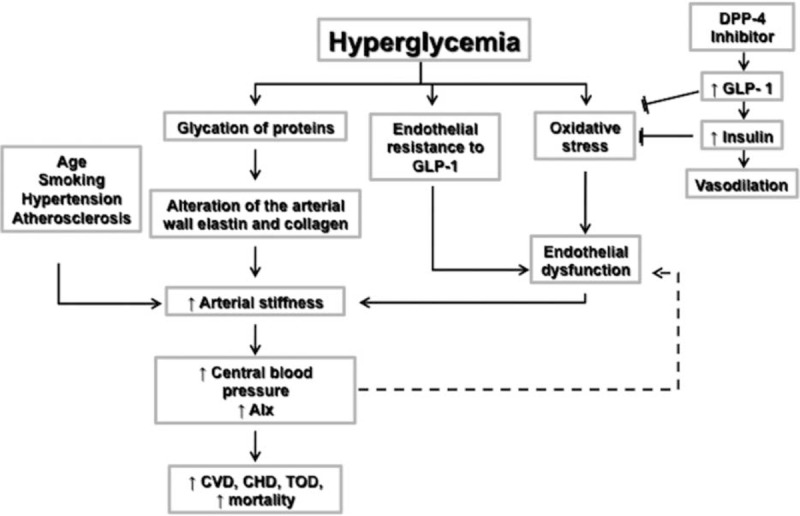
Pathophysiological mechanisms that associate hyperglycemia, endothelial dysfunction, arterial stiffness, and cardiovascular disease. This figure explains how the hyperglycemia causes endothelial damage, which results in arterial stiffness and increased central BP. The GLP-1 system provides beneficial effect on the endothelium in 2 ways, directly in the oxidative stress modulation (blocked arrow) or indirectly in the insulin production increase. The elevated central BP and AIx may interfere in the endothelial dysfunction (dashed arrow), and contribute to the arterial stiffness. AIx = augmentation index, BP = blood pressure, CHD = coronary heart disease, CVD = cardiovascular disease, DPP-4 = dipeptidyl peptidase-4, GLP-1 = glucagon-like peptide 1, TOD = target organ damage.

Although pharmacotherapy based on the GLP-1 system may provide beneficial effects to the endothelium (Figure 2), there are no studies on the central BP. Different methodologies have demonstrated the effect of this pharmacological class on endothelium.^4–6,14,15^ Recently, vildagliptin improved endothelium-dependent vasodilatation in subjects with DM.^5^ However, this study included an invasive method to assess endothelial function, which is not applicable in the clinical practice.^5^ Sitagliptin, another DPP-4 inhibitor, demonstrated the same beneficial effects: an increase in endothelial progenitor cells by inhibiting the degradation of the chemokine stromal-derived factor 1-alpha^4^ and improved endothelial function in uncontrolled diabetic patients.^6^ Some studies on this class of drugs have shown effects on NO modulation.^6,14^ Liraglutide, a new GLP-1 receptor agonist, reduced the plasminogen activator inhibitor 1 and asymmetric dimethylarginine levels and, consequently, improved NO availability.^15^

Several mechanisms may be underlying the improvement in endothelium function linked to vildagliptin use. Firstly, a vasodilator response to acetylcholine was observed in the vascular bed.^5^ In second place, vildagliptin is able to control the daily acute glucose fluctuations and delay the progression of atherosclerosis in DM.^16^ In this study, it was demonstrated that the cIMT, a surrogate of carotid atherosclerosis, decreased 3 months after the use of both vildagliptin and sitagliptin. Finally, GLP-1 has been shown to exert anti-inflammatory effects on different tissues and to decrease daily oxidative stress parameters.^17^ Thus, the decrease in the cIMT might be mediated by improved vascular inflammation and endothelial dysfunction. In the present study, daily acute glucose fluctuations were not evaluated.

To better differentiate the effect of DPP-4 inhibitors on hemodynamic parameters (endothelial function, arterial stiffness, peripheral, and central SBP), it is important to consider the role played by changes in lifestyle on these benchmarks. Some authors showed that individuals treated with DPP-4 inhibitors presented reductions in peripheral SBP independent of decreases in blood glucose and without reducing BMI.^18^ On the contrary, in obese patients with type 2 diabetes, treatment using DPP-4 inhibitors in combination with metformin was associated with improvements in glycemic control and a reduction in body weight.^19^ Thus, it remains to be seen whether the reduction in BP with DPP-4 inhibitor treatment is related to the improvements in blood glucose and the drop in body weight or the effects of this therapeutic drug itself or both.

The effect of weight loss on central BP is controversial. Recently, a study showed that weight loss was significantly and independently associated with central BP and brachial BP. However, the study population included a small number of hypertensive (35.8%) and diabetic (15.8%) patients.^20^ In morbidly obese dysglycemic subjects without hypertension, modest weight loss reduced arterial stiffness.^21^ Another study demonstrated that 10.6% of weight loss did not influence the vascular stiffness in a nondiabetic population.^22^ In obese children and adolescents, no clear effect on arterial stiffness was found in respect to weight reduction.^23^ Moreover, low-fat versus low-carbohydrate diet in adults without DM demonstrated significant weight loss in both groups. However, arterial stiffness improved only in the group following the low-fat diet.^24^ In conclusion, with these data, the effect of weight loss on arterial stiffness is not clear, suggesting that more studies are necessary, including a study with a specific population (hypertensive and DM without coronary disease). In relationship to exercise, there is evidence that resistance training has an effect on the central BP^25^; however, the patient in this case did not do intense exercising. In respect to HbA1c, some studies demonstrated weight loss in adults with DM managed by low-carbohydrate diet, but the HbA1c level decreased only by 0.6% to 1.0%, while the weight loss was between 1.2 and 4.2 kg. These studies justified that weight loss participated in glycemic control, but it was not enough to decrease the HbA1c level.^26,27^

Furthermore, studies have shown solely reduction in peripheral BP with the use of DPP-4 inhibitors, without observing the effects on the central BP. So, we strongly believe that vildagliptin was the main responsible for the improvement in endothelial function, and peripheral and central SBP. Anyway, as we know that one case report is not proof of the effect of DPP-4 inhibitor on central BP, a study that evaluates the endothelial function and glucose-independent beneficial cardiovascular effects of DPP-4 inhibitor is being carried out with this purpose. (J.F. Vilela-Martin, MD, PhD, unpublished data, February 2015, https://clinicaltrials.gov/ct2/show/NCT02145611?term=vildagliptin&rank=8).

## CONCLUSION

In conclusion, to the best of our knowledge this is the first report that suggests an effect of the DPP-4 inhibitor on arterial stiffness parameter (central BP) in a hypertensive and type 2 diabetic individual, using a noninvasive method for evaluating the central BP. Other studies that evaluate the endothelial function and show glucose-independent beneficial cardiovascular effects of DPP-4 inhibitor are expected.
